# Supplementary material for: Axial compression behavior and failure mechanism of CFRP-confined circular hollow steel tube short columns: Theoretical and numerical analysis
Source: PLoS One. 2025 Apr 28;20(4):e0320042. doi: 10.1371/journal.pone.0320042 (PMC12036907; doi:10.1371/journal.pone.0320042)
Supplement: Attachment — Submitted filename: Response to Reviewers.docx [file pone.0320042.s003.docx]

Response to Reviewer Comments Prepared for

**PLOS ONE**

(Manuscript Number: PONE-D-24-42244, January 17, 2025)

Axial compression behavior and failure mechanism of CFRP-confined circular hollow steel tube short columns: theoretical and numerical analysis

Jian Chen ^a^, Hairong Huang ^a^, Yun Zhou ^a, b^, Kan Liu ^c^

^a^ Architectural Engineering Institute, Zhejiang Tongji Vocational College of Science and Technology, China

^b^ College of Water Conservancy and Architectural Engineering, Shihezi University, China

^c^ College of Architecture and Environment, Sichuan University, China

Highlights

- Theoretical formulas for the load-bearing capacity of CFRP-CHST are proposed.
- The failure mechanisms of CFRP-CHST have been extensively analyzed.
- The optimal design scheme for the CFRP-CHST short column is recommended.
- CFRP confinement coefficient optimizes reinforcement and reduces material waste.

**Corresponding author:**

Hairong Huang

Associate professor

Architectural Engineering Institute

Zhejiang Tongji Vocational College of Science and Technology

PR China

E-mail: [z20120100803@zjtongji.edu.cn](mailto:z20120100803@zjtongji.edu.cn) (H.R. Huang)

**Response to Reviewer Comments**

Dear **Reviewers**,

I would like to express sincere gratitude for your insightful and constructive feedback on our manuscript titled "*Axial compression behavior and failure mechanism of CFRP-confined circular hollow steel tube short columns: theoretical and numerical analysis*." Your comprehensive and thoughtful comments have significantly enhanced the depth and scientific rigor of our research. We appreciate your dedication to the peer review process. All your suggestions and concerns have been thoroughly reviewed and addressed. The revised manuscript incorporates these suggestions, with the modified content highlighted in blue for easy reference. Additionally, a detailed response to each comment is provided. Reviewer comments are presented in italics, followed by our corresponding responses. The following are point-by-point revisions made based on your valuable feedback:

1. Response to Reviewer #1

General comments:

This paper presents a study that combines theoretical research with numerical analysis to evaluate the effectiveness of CFRP reinforcement in CHST short columns under axial compression. New formulas for calculating the yield and ultimate bearing capacities of CFRP-CHST short columns are proposed and validated through comparisons with experimental results and finite element analysis. It is essential to highlight the main contributions of this research while clearly differentiating the work conducted by others from the new insights presented in this study. The following issues need to be addressed for clarification.

Response:

Dear Reviewer,

We greatly appreciate your invaluable feedback and the opportunity to enhance the quality of our work. Each of your suggestions has been carefully considered and incorporated into the revised manuscript. We trust that these revisions align with your expectations for publication. Furthermore, we are grateful for your guidance in highlighting the primary contributions of our research and distinguishing our work from previous studies. This study investigates the mechanical behavior and failure mechanisms of CFRP-confined circular hollow steel tube short columns (CFRP-CHST) under axial compression through a combination of theoretical analysis and numerical simulations. The main contributions of the study are outlined as follows:

**1. Development of Theoretical Formulas**

New theoretical formulas have been proposed, based on continuum mechanics and the limit equilibrium method, to calculate the yield and ultimate load-bearing capacities of CFRP-CHST short columns. These formulas offer a solid theoretical foundation for the reinforcement and design of such components in practical engineering applications.

**2. Introduction of the CFRP Confinement Factor**

The concept of the CFRP confinement factor has been introduced to define the effective range of CFRP reinforcement in enhancing the load-bearing capacity of CHST short columns. This provides a quantitative basis for optimizing the number of CFRP layers in engineering design, helping to reduce material waste and improve reinforcement efficiency.

**3. Failure Mechanism Analysis**

A comprehensive analysis of the failure mechanisms of CFRP-CHST short columns was conducted. It was found that although CFRP reinforcement can enhance the ultimate load-bearing capacity within a certain range, this improvement is limited by inward buckling deformation occurring outside the ends of the short columns. This finding offers significant practical insights for the application of CFRP reinforcement.

**4. Optimization of CFRP Layer Number**

The study demonstrated that when the CFRP confinement factor exceeds a certain threshold, increasing the number of CFRP layers does not significantly enhance the ultimate load-bearing capacity of the short column. This insight helps avoid excessive material usage in engineering, making structural design more cost-effective and efficient.

**5. Numerical Analysis Model**

A detailed finite element model was developed, accounting for material, geometric, and contact nonlinearities. The model’s reliability in simulating the failure process and modes of short columns was confirmed through comparisons with experimental results.

Compared to previous studies, this research not only provides a comprehensive approach combining theoretical derivation, numerical simulation, and experimental validation but also introduces the CFRP confinement factor and thoroughly examines its impact on load-bearing capacity and failure modes. This offers new theoretical insights and practical design references for the field, providing significant guidance for future engineering design.

Comment 1:

*Acronyms and abbreviations need to be defined when they are mentioned for the first time. Acronyms are used in many locations without definition. Symbols are used without definition. Please add a* *list for the symbols used.*

Response 1:

Thank you for your valuable feedback. The concerns you raised regarding the definition of abbreviations and symbols are essential for ensuring the clarity and readability of the manuscript. We have incorporated your suggestions, and in the revised manuscript, all abbreviations and symbols are defined upon their first appearance. Additionally, a list summarizing the symbols used has been included. The specific revisions made in the manuscript are as follows:

[Nomenclature, pages 2~ 3; Section 2, Pages 5 ~ 13] A section titled "Nomenclature" has been added before the introduction, providing clear definitions for all abbreviations and acronyms used throughout the manuscript. In addition, below each formula in Section 2, definitions or explanations have been provided for parameters appearing for the first time.

To assist the review process, the newly added section titled "Nomenclature" is provided below:

**Nomenclature**

| Symbol | Meaning | Unit |
| --- | --- | --- |
| CFRP | Carbon fiber-reinforced polymer | - |
| GFRP | Glass fiber-reinforced polymer | - |
| CHST | Circular hollow steel tube | - |
| CFRP-CHST | CFRP-confined CHST short column | - |
| RHST | Rectangular hollow steel tube | - |
| SHST | Square hollow steel tube | - |
| CFST | Concrete-filled steel tube | - |
| FE | Finite element | - |
| $L$ | The length of the CFRP-CHST short column | mm |
| $\sigma_{\text{zs}}$ | Axial stress in the steel tube | MPa |
| $\sigma_{\text{rs}}$ | Radial stress in the steel tube | MPa |
| $\sigma_{\text{θs}}$ | Circumferential stress in the steel tube | MPa |
| $q_{f}$ | Circumferential confinement force provided by CFRP | MPa |
| $t_{s}$ | Thickness of the steel tube | mm |
| $d$ | The inner diameter of the steel tube | mm |
| $D$ | The outer diameter of the steel tube | mm |
| $f_{y}$ | Yield strength of steel | MPa |
| $E_{\text{T,f}}$ | Tensile modulus in the principal fiber direction | MPa |
| $E_{\text{L,f}}$ | Tensile modulus perpendicular to the principal fiber direction | MPa |
| $t_{f}$ | Thickness of the single-layer CFRP | mm |
| $n_{T}$ | Number of winding layers for circumferential fibres | - |
| $n_{L}$ | Number of winding layers for longitudinal fibers | - |
| $t_{\text{eq,f}}$ | Equivalent total thickness of the fibers | mm |
| $N$ | Bearing capacity of CHST | kN |
| $A_{s}$ | The cross-sectional area of the steel tube | $\mathrm{mm}^{2}$ |
| $A_{f}$ | The cross-sectional area of fibres | $\mathrm{mm}^{2}$ |
| $\varepsilon_{\text{θs}}$ | Circumferential strain of steel tube | $\mu\varepsilon$ |
| $E_{\text{s}}$ | Elastic modulus of steel | MPa |
| $\mu_{s}$ | Poisson's ratio of steel | - |
| $\varepsilon_{\text{θ}\text{f}}$ | Circumferential strain of fibers | $\mu\varepsilon$ |
| $\sigma_{\text{θ}\text{f}}$ | Circumferential stress of fibers | MPa |
| $q_{\text{f,y}}$ | Radial stress in the steel tube at yielding | MPa |
| $\psi$, $\beta$, $\gamma$ | Calculation parameters for radial stress | - |
| $\varepsilon_{\text{zs}}$ | Axial strain of steel tube | $\mu\varepsilon$ |
| $\varepsilon_{\text{zs}\text{,y}}$ | Axial yield strain of the steel tube | $\mu\varepsilon$ |
| $\Gamma$ | Calculation parameters for strain | - |
| $\varepsilon$ | Axial compressive strain | $\mu\varepsilon$ |
| $\Delta L$ | The axial compression displacement of the component. | mm |
| $L$ | Total length of the component | mm |
| $\Delta l_{\text{sf,y}}$ | The theoretical value of the axial displacement at the yield point of the CFRP-CHST short column | mm |
| $\Delta l_{\text{FE,y}}$ | The finite element value of the axial displacement at the yield point of the CFRP-CHST short column | mm |
| $q_{\text{f}\text{,y}}$ | Radial stress of the steel tube at the yield state | MPa |
| $q_{\text{f,}\text{l}}$ | Radial stress of steel tube at the ultimate state | MPa |
| $f_{f}$ | The ultimate strength of the fibers | MPa |
| $\xi_{f}$ | CFRP confinement coefficient | - |
| $W_{\text{sf,y}}$ | Improvement ratio of the yield load-bearing capacity | % |
| $W_{\text{sf,}\text{l}}$ | Improvement ratio of the ultimate load-bearing capacity | % |
| $N_{\text{s,y}}$ | Yield bearing capacity for CHST short columns | kN |
| $N_{\text{FE,y}}$ | Finite element value of the yield load-bearing capacity of CFRP-CHST short columns | kN |
| $N_{\text{sf,y}}$ | The theoretical value of yield-bearing capacity of CFRP-CHST short columns | kN |
| $N_{e\text{,}\text{l}}$ | The experimental value of the ultimate load-bearing capacity of CFRP-CHST short columns | kN |
| $N_{\text{sf,l}}$ | The theoretical value of the ultimate load-bearing capacity of CFRP-CHST short columns | kN |
| $N_{\text{FE,l}}$ | Finite element value of ultimate load-bearing capacity of CFRP-CHST short columns | kN |

Comment 2:

Please include an illustration for cross-sectional information (D, d, ts, and L) in Fig 1.

Response 2:

The authors express their sincere gratitude to the reviewers for their suggestions regarding the annotation of cross-sectional dimensions. In response to the reviewers' comments, Figure 1 now clearly illustrates the outer diameter ($D$) and inner diameter ($d$) of the circular hollow steel tube, the wall thickness ($t_{s}$) of the steel tube, and the length ($L$) of the specimen. The specific revisions made in the manuscript are as follows:

[Figure 1, Page 6] Figure 1 has been updated to include more detailed dimension annotations, as suggested by the reviewer.

Additionally, to facilitate the review process, the schematic diagram of the modified CFRP-CHST short column is provided in Fig. R1- 1.

Fig. R1- 1 CFRP-CHST short column: (a) elevation, and (b) cross-section.

Comment 3:

On page 12, the authors summarized the specimens tested by Haedir and Zhao [18], but they did not provide any information about the specimens used in the experiments conducted by Teng and Hu [23]. A Comprehensive Table Considering All Specimens Should Be Included. Additionally, Tables 4 and 5 do not include comparisons of the specimens tested by Teng and Hu [23].

Response 3:

We appreciate your attention to detail in our manuscript. Your feedback is highly valuable, and we agree that including a comprehensive table detailing the sample information from the experiments conducted by Teng and Hu [2] is essential. In response to your feedback, Table 1 has been expanded to include a comprehensive list of all specimens from Haedir and Zhao [1] as well as Teng and Hu [2]. This table now provides detailed parameters for each sample, enabling readers to compare material properties, dimensions, and CFRP layer configurations across all experiments. Furthermore, Tables 4 and 5 have been updated to include the test results of the specimens from Teng and Hu [2], allowing for a more thorough comparison. The specific revisions in the manuscript are as follows:

[Table 1, Table 2, Table 3] The original Table 1, Table 4, and Table 5 have been revised in the updated manuscript and are now designated as Table 1, Table 2, and Table 3, respectively.

[1]	J. Haedir, X.-L. Zhao, Design of short CFRP-reinforced steel tubular columns, Journal of Constructional Steel Research 67 (2011) 497–509. https://doi.org/10.1016/j.jcsr.2010.09.005.

[2]	J.G. Teng, Y.M. Hu, Behaviour of FRP-jacketed circular steel tubes and cylindrical shells under axial compression, Construction and Building Materials 21 (2007) 827–838. https://doi.org/10.1016/j.conbuildmat.2006.06.016.

To facilitate your review process, the revised versions of Table 1, Table 2, and Table 3 are provided in Table R1- 1, Table R1- 2, and Table R1- 3, respectively.

Table R1- 1 Model parameters

| Ref | Group | Specimen ID | $D$ /mm | $t_{s}$/mm | $n_{H}$ | $n_{L}$ |
| --- | --- | --- | --- | --- | --- | --- |
| [19] | 1 | C-1 | 87.25 | 2.36 | 0 | 0 |
|  |  | CF-1A | 87.23 | 2.32 | 1 | 1 |
|  |  | CF-1B | 87.21 | 2.32 | 2 | 2 |
|  | 2 | C-2 | 86.31 | 2 | 0 | 0 |
|  |  | CF-2A | 86.38 | 1.96 | 1 | 1 |
|  |  | CF-2B | 86.39 | 1.96 | 2 | 2 |
|  | 3 | C-3 | 85.75 | 1.57 | 0 | 0 |
|  |  | CF-3A | 85.68 | 1.57 | 1 | 1 |
|  | 4 | C-4 | 85.11 | 1.13 | 0 | 0 |
|  |  | CF-4A | 85.21 | 1.1 | 1 | 1 |
| [24] | 5 | ST1 | 165 | 4.2 | 0 | 0 |
|  |  | ST2 | 166 | 4.2 | 1 | 0 |
|  |  | ST3 | 165 | 4.2 | 2 | 0 |
|  |  | ST4 | 165 | 4.2 | 3 | 0 |

Note: $D$ is the outer diameter of the steel tube, $t_{s}$ is the thickness of the steel tube, $n_{H}$ is the number of winding layers for circumferential fibres, and $n_{L}$ is the number of winding layers for longitudinal fibers.

Table R1- 2 Comparison of longitudinal displacement at yield for CHST short columns

| Ref | Group | Specimen ID | $L$ /mm | $\varepsilon_{\text{zs,y}}$/$\text{με}$ | $\Delta l_{\text{sf,y}}$/mm | $\Delta l_{\text{FE,y}}$/mm | $\frac{\Delta l_{\text{sf,y}}}{\Delta l_{\text{FE,y}}}$ |
| --- | --- | --- | --- | --- | --- | --- | --- |
| [19] | 1 | C-1 | 275 | 2171 | 0.5971 | 0.6183 | 0.97 |
|  |  | CF-1A | 275 | 2183 | 0.6004 | 0.6217 | 0.97 |
|  |  | CF-1B | 275 | 2197 | 0.6042 | 0.6254 | 0.97 |
|  | 2 | C-2 | 275 | 2171 | 0.5971 | 0.6267 | 0.95 |
|  |  | CF-2A | 275 | 2186 | 0.6010 | 0.6307 | 0.95 |
|  |  | CF-2B | 275 | 2202 | 0.6055 | 0.6352 | 0.95 |
|  | 3 | C-3 | 275 | 2171 | 0.5971 | 0.6219 | 0.96 |
|  |  | CF-3A | 275 | 2189 | 0.6021 | 0.6269 | 0.96 |
|  | 4 | C-4 | 275 | 2171 | 0.5971 | 0.6198 | 0.96 |
|  |  | CF-4A | 275 | 2197 | 0.6043 | 0.6270 | 0.96 |
| [24] | 5 | ST1 | 450 | 1660 | 0.7469 | 0.7765 | 0.96 |
|  |  | ST2 | 450 | 1661 | 0.7476 | 0.7765 | 0.96 |
|  |  | ST3 | 450 | 1663 | 0.7484 | 0.7766 | 0.96 |
|  |  | ST4 | 450 | 1665 | 0.7492 | 0.7766 | 0.96 |
| Average |  |  |  |  |  |  | 0.96 |
| Standard deviation |  |  |  |  |  |  | 0.0048 |

Note: $L$ is the length of the CFRP-CHST short column, $\varepsilon_{\text{zs,y}}$ is axial yield strain of the steel tube, $\Delta l_{\text{sf,y}}$ is the theoretical value of the axial displacement at the yield point of the CFRP-CHST short column, $\Delta l_{\text{FE,y}}$ is the Finite element value of the axial displacement at the yield point of the CFRP-CHST short column.

Table R1- 3 Comparison of theoretical, experimental, and finite element values for yield and ultimate load-bearing capacity

| Ref | Group | Specimen ID | $t_{s}$/  mm | $N_{\text{s,y}}$/  kN | Yield load-bearing capacity | | | | | Ultimate load-bearing capacity | | | | | | | |
| --- | --- | --- | --- | --- | --- | --- | --- | --- | --- | --- | --- | --- | --- | --- | --- | --- | --- |
|  |  |  |  |  | $q_{\text{f,y}}$/  MPa | $N_{\text{sf,y}}$/  kN | $W_{\text{sf,y}}$/  % | $N_{\text{FE,y}}$/  kN | $\frac{N_{\text{sf,y}}}{N_{\text{FE,y}}}$ | $q_{\text{f,l}}$/  MPa | $N_{\text{sf,l}}$/  kN | $W_{\text{sf,l}}$/  % | $N_{\text{FE,l}}$/  kN | $N_{\text{e,l}}$/  kN | $\frac{N_{\text{sf,l}}}{N_{\text{e,l}}}$ | $\frac{N_{\text{sf,l}}}{N_{\text{FE,l}}}$ | $\frac{N_{\text{FE,l}}}{N_{\text{e,l}}}$ |
| [19] | 1 | C-1 | 2.36 | 286 | 0.0 | 286 | 0.00 | 280 | 1.02 | 0.0 | 286 | 0.00 | 280 | 253 | 1.13 | 1.02 | 1.11 |
|  |  | CF-1A | 2.32 | 281 | 0.7 | 286 | 1.45 | 285 | 1.00 | 7.4 | 317 | 12.51 | 312 | 299 | 1.06 | 1.01 | 1.04 |
|  |  | CF-1B | 2.32 | 281 | 1.6 | 290 | 3.18 | 287 | 1.01 | 14.9 | 334 | 18.70 | 330 | 341 | 0.98 | 1.01 | 0.97 |
|  | 2 | C-2 | 2 | 241 | 0.0 | 241 | 0.00 | 235 | 1.03 | 0.0 | 241 | 0.00 | 235 | 220 | 1.10 | 1.03 | 1.07 |
|  |  | CF-2A | 1.96 | 236 | 0.7 | 240 | 1.73 | 240 | 1.00 | 7.5 | 270 | 14.01 | 261 | 267 | 1.01 | 1.03 | 0.98 |
|  |  | CF-2B | 1.96 | 236 | 1.7 | 246 | 3.86 | 243 | 1.01 | 15.0 | 280 | 18.34 | 273 | 281 | 1.00 | 1.02 | 0.97 |
|  | 3 | C-3 | 1.57 | 189 | 0.0 | 189 | 0.00 | 183 | 1.03 | 0.0 | 189 | 0.00 | 183 | 170 | 1.11 | 1.03 | 1.08 |
|  |  | CF-3A | 1.57 | 189 | 0.8 | 193 | 2.19 | 189 | 1.02 | 7.6 | 219 | 15.84 | 210 | 214 | 1.02 | 1.04 | 0.98 |
|  | 4 | C-4 | 1.13 | 136 | 0.0 | 136 | 0.00 | 131 | 1.03 | 0.0 | 136 | 0.00 | 131 | 120 | 1.13 | 1.03 | 1.09 |
|  |  | CF-4A | 1.1 | 132 | 0.8 | 137 | 3.27 | 134 | 1.02 | 7.6 | 155 | 17.06 | 148 | 155 | 1.00 | 1.05 | 0.95 |
| [24] | 5 | ST1 | 4.2 | 707 | 0.0 | 707 | 0.00 | 710 | 1.00 | 0.0 | 707 | 0.00 | 721 | 718 | 0.99 | 0.98 | 1.00 |
|  |  | ST2 | 4.2 | 712 | 0.1 | 714 | 0.26 | 724 | 0.99 | 3.7 | 780 | 9.58 | 745 | 740 | 1.05 | 1.05 | 1.01 |
|  |  | ST3 | 4.2 | 707 | 0.2 | 711 | 0.52 | 735 | 0.97 | 7.5 | 821 | 16.03 | 768 | 771 | 1.06 | 1.07 | 1.00 |
|  |  | ST4 | 4.2 | 707 | 0.3 | 713 | 0.79 | 743 | 0.96 | 11.3 | 841 | 18.83 | 803 | 782 | 1.07 | 1.05 | 1.03 |
| Average | | |  |  |  |  |  |  | 1.01 |  |  |  |  |  | 1.05 | 1.03 | 1.02 |
| Standard deviation | | |  |  |  |  |  |  | 0.0227 |  |  |  |  |  | 0.0532 | 0.0213 | 0.0535 |

Note: $t_{s}$ is the thickness of the steel tube, $N_{\text{s,y}}$ is the yield-bearing capacity for CHST short columns, $q_{\text{f}\text{,y}}$ is radial stress of the steel tube at the yield state, $N_{\text{sf,y}}$ is the theoretical value of the yield-bearing capacity of CFRP-CHST short columns, $W_{\text{sf,y}}$ is the improvement ratio of the yield load-bearing capacity, $N_{\text{FE,y}}$ is the finite element value of the yield load-bearing capacity of CFRP-CHST short columns, $q_{\text{f,l}}$ is radial stress of steel tube at the ultimate state, $N_{\text{sf,l}}$ is the theoretical value of the ultimate load-bearing capacity of CFRP-CHST short columns, $W_{\text{sf,l}}$ is the improvement ratio of the ultimate load-bearing capacity, $N_{\text{FE,l}}$ is the finite element value of the ultimate load-bearing capacity of CFRP-CHST short columns, $N_{\text{e,l}}$ is the experimental value of the ultimate load-bearing capacity of CFRP-CHST short columns.

Comment 4:

It is imperative to include the specified parameters and to provide a clear rationale for their selection, along with a detailed explanation of their anticipated impact on the behavior of circular CFRP-CHST short columns. This will significantly enhance understanding. The parametric study must encompass a comprehensive range of parameters to thoroughly examine their effects on theoretical formula of the yield and ultimate bearing capacities of CFRP-CHST short columns.

Response 4:

The author greatly appreciates the reviewer’s suggestions and agrees that a comprehensive parametric study would enhance the understanding of CFRP-CHST short columns' behavior, providing valuable guidance for engineering design. In response to your suggestion, a parametric analysis section has been added to the revised manuscript, along with justifications for the selection of parameters. Additionally, a detailed analysis of the impact of key design parameters on the behavior of CFRP-CHST short columns has been conducted, with particular emphasis on their effects on yield and ultimate load-bearing capacity.

**1. Selection of Key Parameters**

Six key parameters were selected for the analysis: the thickness of the steel tube ($t_{s}$), the number of CFRP layers ($n_{T}$ and $n_{L}$), the yield strength of the steel tube ($f_{y}$), the tensile strength of the CFRP ($f_{f}$), the diameter-to-thickness ratio of the steel tube ($D/{t_{s}}$), and the CFRP confinement coefficient ($\xi_{f}$). The rationale for selecting these parameters is as follows:

- $t_{s}$ directly affects the local buckling and overall stability of the short column.
- $n_{T}$ and $n_{L}$ are critical for determining the strengthening effect of the short column.
- $f_{y}$ and $f_{f}$ are essential in assessing the structural response under axial loading.
- The diameter-to-thickness ratio of the steel tube ($D/{t_{s}}$) plays a significant role in determining the load-bearing capacity and buckling behavior of the CFRP- CHST short column.
- $\xi_{f}$ defines the effective range of CFRP strengthening.

**2. Influence of Key Parameters on the Load-Bearing Capacity of Short Columns**

A total of 32 short column specimens with varying geometric and material parameters were analyzed using the validated theoretical formulas. The specific model parameters are presented in Table R1- 4. A detailed discussion of the influence of the key design parameters ($t_{s}$, $n_{T}$, $n_{L}$, $f_{y}$, $f_{f}$, $D/{t_{s}}$, and $\xi_{f}$) on the yield and ultimate load-bearing capacity of CFRP-CHST short columns follows.

**2.1 Thickness of the steel tube (**$\boldsymbol{t}_{\mathbf{s}}$**)**

Fig. R1- 2 (a) and Fig. R1- 2 (b) show the variation in the yield and ultimate load-bearing capacities of CFRP-CHST short columns with changes in steel tube thickness. As illustrated in the figures, both the yield and ultimate load-bearing capacities increased with the steel tube thickness, demonstrating a clear linear relationship.

Fig. R1- 2 Effect of the steel tube thickness on load-bearing capacity: (a) yield load-bearing capacity, and (b) ultimate load-bearing capacity.

**2.2 Yield strength of the steel tube (**$\boldsymbol{f}_{\mathbf{y}}$**)**

Fig. R1- 3 (a) and Fig. R1- 3 (b) illustrate the variation trends of the yield and ultimate load-bearing capacities of CFRP-CHST short columns under different steel tube yield strengths. The data points in the figures display a clear linear growth trend, indicating a positive correlation between the increase in steel tube yield strength and both the yield and ultimate load-bearing capacities of the component.

Fig. R1- 3 Effect of steel yield strength on load-bearing capacity: (a) yield load-bearing capacity, and (b) ultimate load-bearing capacity.

**2.3 Number of CFRP layers (**$\boldsymbol{n}_{\mathbf{T}}$ **and** $\boldsymbol{n}_{\mathbf{L}}$**)**

Fig. R1- 4 (a) illustrates the variation trend of the yield load-bearing capacity of CFRP-CHST short columns with different numbers of CFRP layers. The data points in the figure show an approximately linear growth trend, indicating a positive correlation between the increase in the number of CFRP layers and the yield load-bearing capacity of the component. This is likely due to the additional confinement provided by extra CFRP layers, which enhances the stiffness and strength of the component. Fig. R1- 4 (b) demonstrates the effect of the number of CFRP layers on the ultimate load-bearing capacity of CFRP-CHST short columns. A nonlinear growth trend is observed, suggesting that the rate of increase in ultimate load-bearing capacity gradually slows as the number of CFRP layers increases. The data points stabilize after reaching a certain number of layers, indicating the presence of a saturation point. Beyond this point, increasing the number of CFRP layers has a limited effect on further enhancing the ultimate load-bearing capacity. This saturation effect provides a basis for optimizing the number of CFRP layers, helping to avoid unnecessary material usage. When designing CFRP-CHST short columns, cost-effectiveness should be considered, and an appropriate number of CFRP layers should be selected to achieve both optimal ultimate load-bearing capacity and economic efficiency.

Fig. R1- 4 Effect of the number of CFRP layers on load-bearing capacity: (a) yield load-bearing capacity, and (b) ultimate load-bearing capacity.

**2.4 Tensile strength of the CFRP (**$\boldsymbol{f}_{\mathbf{f}}$**)**

Fig. R1- 5 (a) illustrates the variation trend of the yield load-bearing capacity of CFRP-CHST short columns with different CFRP tensile strengths. The data points in the figure appear nearly horizontal, indicating that, within the tested tensile strength range, the CFRP tensile strength has a negligible effect on the yield load-bearing capacity of the component. Fig. R1- 5 (b) shows the effect of CFRP tensile strength on the ultimate load-bearing capacity of CFRP-CHST short columns. The data points in the figure display a clear nonlinear growth trend, indicating a positive correlation between the increase in CFRP tensile strength and the ultimate load-bearing capacity of the component.

Fig. R1- 5 Effect of CFRP tensile strength on load-bearing capacity: (a) yield load-bearing capacity, and (b) ultimate load-bearing capacity.

**2.5 Diameter-to-thickness ratio of the steel tube (**$\boldsymbol{D}/{\boldsymbol{t}_{\mathbf{s}}}$**)**

Fig. R1- 6 (a) and Fig. R1- 6 (b) illustrate the variation trends of the yield and ultimate load-bearing capacities of CFRP-CHST short columns under different diameter-to-thickness ratios. The data points in the figures exhibit a clear nonlinear decreasing trend, indicating that as the diameter-to-thickness ratio increases, both the yield and ultimate load-bearing capacities of the component significantly decrease. In the range of smaller diameter-to-thickness ratios, the load-bearing capacity decreases rapidly. However, as the diameter-to-thickness ratio increases, the rate of decrease gradually slows down. An increase in the steel tube diameter-to-thickness ratio may reduce the geometric stability of the component, thereby lowering its load-bearing capacity. When designing CFRP-CHST short columns, the diameter-to-thickness ratio should be carefully considered to ensure that the component maintains adequate yield load-bearing capacity.

Fig. R1- 6 Effect of the diameter-to-thickness ratio of the steel pipe on load-bearing capacity: (a) yield load-bearing capacity, and (b) ultimate load-bearing capacity.

**2.6 CFRP confinement coefficient (**$\boldsymbol{\xi}_{\mathbf{f}}$**)**

Fig. R1- 7 (a) illustrates the variation trend of the yield load-bearing capacity of CFRP-CHST short columns under different CFRP confinement coefficients. The data points in the figure show an approximately linear growth trend, indicating a positive correlation between the increase in the CFRP confinement coefficient and the yield load-bearing capacity of the component. As the CFRP confinement coefficient increases, the yield load-bearing capacity of the CFRP-CHST short columns is enhanced. In contrast, Fig. R1- 7 (b) exhibits a nonlinear growth trend, indicating that the rate of increase in the ultimate load-bearing capacity gradually slows down as the CFRP confinement coefficient increases. The data points stabilize after reaching a certain confinement coefficient, suggesting the presence of a saturation point. Beyond this point, further increases in the CFRP confinement coefficient have a limited effect on enhancing the ultimate load-bearing capacity. This observation provides a basis for optimizing the CFRP confinement coefficient to avoid unnecessary material waste. When designing CFRP-CHST short columns, cost-effectiveness should be considered, and an appropriate CFRP confinement coefficient should be selected to achieve both optimal ultimate load-bearing capacity and economic efficiency.

Fig. R1- 7 Effect of CFRP confinement coefficient on load-bearing capacity: (a) yield load-bearing capacity, and (b) ultimate load-bearing capacity.

The above discussion has been fully incorporated into the revised manuscript to enhance the comprehensiveness of the study. It is believed that this comprehensive parametric study offers a better understanding of the influence of various parameters on the behavior of CFRP-CHST short columns, thereby providing more accurate guidance for engineering design.

The specific revisions in the manuscript are as follows:

[Section 6, pages 26 ~ 30; Appendix A, Pages 33 ~ 34] In the revised manuscript, Section 6 has been added, providing a comprehensive and in-depth discussion of the influence of key parameters on the load-bearing capacity. Additionally, samples of the parameter analysis are provided in Appendix A, as shown in Table R1- 4.

Table R1- 4 Parameter analysis samples

| Specimen ID | Parameters of specimens | | | | | | | | | | | | Bearing capacity | |
| --- | --- | --- | --- | --- | --- | --- | --- | --- | --- | --- | --- | --- | --- | --- |
|  | $D$ /mm | $t_{s}$/mm | $E_{s}$/MPa | $\mu_{s}$ | $f_{y}$/MPa | $E_{\text{T,f}}$/MPa | $t_{f}$/mm | $f_{f}$/MPa | $D/{t_{s}}$ | $\xi_{f}$ | $n_{H}$ | $n_{L}$ | $N_{\text{sf,y}}$/kN | $N_{\text{sf,l}}$/kN |
| PA-1 | 150 | 1.4 | 209571 | 0.3 | 455 | 230000 | 0.176 | 2000 | 107 | 1.11 | 2 | 2 | 314 | 276 |
| PA-2 | 150 | 1.8 | 209571 | 0.3 | 455 | 230000 | 0.176 | 2000 | 83 | 0.87 | 2 | 2 | 397 | 432 |
| PA-3 | 150 | 2.2 | 209571 | 0.3 | 455 | 230000 | 0.176 | 2000 | 68 | 0.71 | 2 | 2 | 480 | 544 |
| PA-4 | 150 | 2.6 | 209571 | 0.3 | 455 | 230000 | 0.176 | 2000 | 58 | 0.60 | 2 | 2 | 562 | 644 |
| PA-5 | 150 | 3 | 209571 | 0.3 | 455 | 230000 | 0.176 | 2000 | 50 | 0.52 | 2 | 2 | 645 | 737 |
| PA-6 | 150 | 3.4 | 209571 | 0.3 | 455 | 230000 | 0.176 | 2000 | 44 | 0.46 | 2 | 2 | 727 | 827 |
| PA-7 | 150 | 3.8 | 209571 | 0.3 | 455 | 230000 | 0.176 | 2000 | 39 | 0.41 | 2 | 2 | 808 | 915 |
| PA-8 | 150 | 4.2 | 209571 | 0.3 | 455 | 230000 | 0.176 | 2000 | 36 | 0.37 | 2 | 2 | 889 | 1001 |
| PA-9 | 150 | 4.6 | 209571 | 0.3 | 455 | 230000 | 0.176 | 2000 | 33 | 0.34 | 2 | 2 | 970 | 1085 |
| PA-10 | 150 | 5 | 209571 | 0.3 | 455 | 230000 | 0.176 | 2000 | 30 | 0.31 | 2 | 2 | 1050 | 1169 |
| PA-11 | 150 | 5 | 209571 | 0.3 | 455 | 230000 | 0.176 | 2000 | 30 | 0.16 | 1 | 1 | 1043 | 1109 |
| PA-12 | 150 | 5 | 209571 | 0.3 | 455 | 230000 | 0.176 | 2000 | 30 | 0.31 | 2 | 2 | 1050 | 1169 |
| PA-13 | 150 | 5 | 209571 | 0.3 | 455 | 230000 | 0.176 | 2000 | 30 | 0.47 | 3 | 3 | 1058 | 1212 |
| PA-14 | 150 | 5 | 209571 | 0.3 | 455 | 230000 | 0.176 | 2000 | 30 | 0.62 | 4 | 4 | 1066 | 1238 |
| PA-15 | 150 | 5 | 209571 | 0.3 | 455 | 230000 | 0.176 | 2000 | 30 | 0.78 | 5 | 5 | 1076 | 1244 |
| PA-16 | 150 | 5 | 209571 | 0.3 | 455 | 230000 | 0.176 | 2000 | 30 | 0.94 | 6 | 6 | 1086 | 1222 |
| PA-17 | 150 | 5 | 209571 | 0.3 | 150 | 230000 | 0.176 | 2000 | 30 | 0.95 | 2 | 2 | 346 | 402 |
| PA-18 | 150 | 5 | 209571 | 0.3 | 200 | 230000 | 0.176 | 2000 | 30 | 0.71 | 2 | 2 | 461 | 547 |
| PA-19 | 150 | 5 | 209571 | 0.3 | 250 | 230000 | 0.176 | 2000 | 30 | 0.57 | 2 | 2 | 577 | 676 |
| PA-20 | 150 | 5 | 209571 | 0.3 | 300 | 230000 | 0.176 | 2000 | 30 | 0.47 | 2 | 2 | 692 | 800 |
| PA-21 | 150 | 5 | 209571 | 0.3 | 350 | 230000 | 0.176 | 2000 | 30 | 0.41 | 2 | 2 | 808 | 921 |
| PA-22 | 150 | 5 | 209571 | 0.3 | 400 | 230000 | 0.176 | 2000 | 30 | 0.35 | 2 | 2 | 923 | 1039 |
| PA-23 | 150 | 5 | 209571 | 0.3 | 450 | 230000 | 0.176 | 2000 | 30 | 0.32 | 2 | 2 | 1038 | 1157 |
| PA-24 | 150 | 5 | 209571 | 0.3 | 500 | 230000 | 0.176 | 2000 | 30 | 0.28 | 2 | 2 | 1154 | 1274 |
| PA-25 | 150 | 5 | 209571 | 0.3 | 550 | 230000 | 0.176 | 2000 | 30 | 0.26 | 2 | 2 | 1269 | 1390 |
| PA-26 | 150 | 5 | 209571 | 0.3 | 600 | 230000 | 0.176 | 2000 | 30 | 0.24 | 2 | 2 | 1384 | 1506 |
| PA-27 | 150 | 5 | 209571 | 0.3 | 350 | 230000 | 0.176 | 1000 | 30 | 0.20 | 2 | 2 | 808 | 868 |
| PA-28 | 150 | 5 | 209571 | 0.3 | 350 | 230000 | 0.176 | 1500 | 30 | 0.30 | 2 | 2 | 808 | 897 |
| PA-29 | 150 | 5 | 209571 | 0.3 | 350 | 230000 | 0.176 | 2000 | 30 | 0.41 | 2 | 2 | 808 | 921 |
| PA-30 | 150 | 5 | 209571 | 0.3 | 350 | 230000 | 0.176 | 2500 | 30 | 0.51 | 2 | 2 | 808 | 939 |
| PA-31 | 150 | 5 | 209571 | 0.3 | 350 | 230000 | 0.176 | 3000 | 30 | 0.61 | 2 | 2 | 808 | 951 |
| PA-32 | 150 | 5 | 209571 | 0.3 | 350 | 230000 | 0.176 | 3500 | 30 | 0.71 | 2 | 2 | 808 | 957 |

Note: $D$ is the outer diameter of the steel tube, $t_{s}$ is the thickness of the steel tube, $E_{s}$ is the elastic modulus of steel, $\mu_{s}$ is the Poisson's ratio of steel, $f_{y}$ is the yield strength of steel, $E_{\text{T,f}}$ is tensile modulus in the principal fiber direction, $t_{f}$ is the thickness of the single-layer CFRP, $f_{f}$ is the ultimate strength of the fibers, $\xi_{f}$ is the CFRP confinement coefficient, $n_{H}$ is the number of winding layers for circumferential fibres, $n_{L}$ is the number of winding layers for longitudinal fibers, $N_{\text{sf,y}}$ is the theoretical value of the yield-bearing capacity of CFRP-CHST short columns, $N_{\text{sf,l}}$ is the theoretical value of the ultimate load-bearing capacity of CFRP-CHST short columns.

Comment 5:

How interaction between the steel and CFRP was modelled?

Response 5:

Thank you for your inquiry regarding the interaction model between steel and CFRP in our study. In the refined finite element model, binding constraints (tie) are applied between the steel tube and CFRP, as well as between the CFRP layers, to ensure their collaborative deformation. A detailed explanation is provided below:

**1. Bonding Assumption**

Experimental studies indicate that CFRP and the steel tube can deform together during loading, with no delamination occurring before failure [1,2]. In our finite element model, we assume perfect bonding between the steel tube and CFRP, meaning no relative slippage occurs at the interface during loading. Binding constraints (tie) are applied between the steel tube and CFRP, as well as between the CFRP layers, to ensure their collaborative deformation.

**2. Numerical Validation**

To validate the finite element (FE) modeling approach, we compared the load-displacement curves and failure modes from the FE model with experimental results. The load-displacement curves and failure modes obtained from the FE model match well with the experimental observations, confirming the effectiveness of our interaction modeling approach.

In summary, the modeling approach for the interaction between steel and CFRP in CFRP-confined CHST short columns is comprehensive, considering both material behavior and bonding conditions. We hope this explanation addresses your question and provides the clarity you sought.

The specific revisions in the manuscript are as follows:

[Section 3, pages 16 ~ 17, lines 307 ~ 312] In Section 3 of the revised manuscript, we provide a detailed description of the interaction between CFRP and steel, clarifying the method used and its underlying principles.

[1]	J. Haedir, X.-L. Zhao, Design of short CFRP-reinforced steel tubular columns, Journal of Constructional Steel Research 67 (2011) 497–509. https://doi.org/10.1016/j.jcsr.2010.09.005.

[2]	J.G. Teng, Y.M. Hu, Behaviour of FRP-jacketed circular steel tubes and cylindrical shells under axial compression, Construction and Building Materials 21 (2007) 827–838. https://doi.org/10.1016/j.conbuildmat.2006.06.016.

Comment 6:

Line 240-241, “the two loading plates at the ends are modeled as analytical rigid bodies”. Please explain in the manuscript.

Response 6:

Thank you for raising the question regarding the modeling of the loading plates as rigid bodies in our analysis. This study focuses on the mechanical behavior and failure mechanisms of CFRP-CHST under axial compression. To achieve this, it is essential to accurately model the interaction between the steel column and CFRP, as well as their response to axial loading. In the experiments, the loading plates are rigid structures used to apply axial compressive forces. Since the stiffness of the loading plates is much greater than that of the steel tube, it is reasonable to assume that the loading plates undergo negligible deformation during the loading process. Moreover, the primary function of the loading plates is to transfer the load, not to bear it. As a result, their internal stress and deformation are not critical for understanding the mechanical behavior of CHST short columns. Based on these considerations, the loading plates at both ends were modeled as rigid bodies. This approach offers several advantages:

**1. Simplified Model**

Treating the loading plates as rigid bodies reduces the model's degrees of freedom, which decreases computational resource requirements without compromising the capture of key responses.

**2. Improved Computational Efficiency**

Rigid body modeling eliminates the need for complex meshing, thereby reducing the complexity of the finite element model and improving computational efficiency.

**3. Accurate Load Transfer**

The rigid body model ensures that the load is accurately transferred from the loading plates to the steel column, without being affected by any deformation of the loading plates themselves.

We recognize that this approach requires further explanation to help readers understand our modeling choice and rationale. As such, we have added a detailed explanation in the revised manuscript to ensure clarity regarding our modeling approach and analysis process. The specific revisions in the manuscript are as follows:

[Section 3, page 15, lines 276 ~ 283] A detailed explanation of the principles behind using rigid body loading plates and the impact of this modeling choice on the overall analysis has been included.

Comment 7:

The study only considers CFRP-CHST short columns under axial compression, not addressing other loading conditions such as flexure or cyclic loading. Mention the limitations regarding loading conditions explicitly and propose future studies to include other loading conditions. Highlight the potential impact of these conditions on the behavior of CFRP-CHST short columns.

Response 7:

Thank you for your suggestion. We fully agree that it is important to clearly state that our study is limited to CFRP-CHST short columns under axial compression and does not address other loading conditions, such as bending or cyclic loading. We will explicitly mention this limitation in the Conclusion section and propose future research directions to explore these other loading conditions. Below is a specific response to this issue:

**1. Limitation of Loading Conditions**

This study focuses on the mechanical behavior and failure mechanisms of CFRP-CHST short columns under axial compression. This specific focus allows for an in-depth analysis of their behavior under a common loading condition. However, we acknowledge that this restricts the applicability of our findings to other loading scenarios, such as bending or cyclic loading, which may significantly impact the stress-strain relationship, local buckling, and overall stability of the columns. Therefore, we clearly state that the conclusions drawn from this study may not apply to these other loading conditions.

**2. Future Research Directions**

To address these limitations and expand the understanding of CFRP-CHST short columns under various loading conditions, we suggest the following future research directions:

- Bending Loading: Investigate the behavior of CFRP-CHST short columns under bending loads, particularly how CFRP confinement enhances the bending performance. This would involve developing new models and simulations to predict bending capacity and deformation, considering the interaction between CFRP and steel under bending conditions. Further, the effect of CFRP layer orientation and the number of layers on bending resistance should be explored.
- Cyclic Loading: Study the performance of CFRP-CHST short columns under cyclic loading, including effects such as fatigue and damage accumulation. This is especially relevant for structures subjected to seismic activity or repeated stress cycles. Future studies could focus on how cyclic loading influences the bond between CFRP and steel, the fatigue life of the columns, and the degradation of material properties over time.
- Combined Loading: Examine the behavior of CFRP-CHST short columns under multi-axial loading conditions, such as axial compression combined with bending or torsion. These combined loadings are common in real-world structures and can affect the load-bearing capacity and failure mechanisms of the columns. Research could develop models to predict the interactions between axial, bending, and torsional stresses and how CFRP confinement influences column stability under such complex loading conditions.
- Long-Term Performance and Durability: Assess the long-term performance and durability of CFRP-CHST short columns under sustained loading and environmental factors such as temperature variations, humidity, corrosion, and UV exposure. This research would provide valuable insights into the behavior of these columns under real-world service conditions and guide design practices for improved durability and reliability.

**3. Potential Impacts**

Other loading conditions may have the following impacts on CFRP-CHST short columns:

- Bending and Cyclic Loading: These conditions could lead to more complex stress distributions, influencing the effectiveness of CFRP confinement. Under bending, the column may experience flexural stress and potential yielding or buckling. CFRP confinement could prevent local buckling but may experience delamination due to shear stresses from bending. Cyclic loading can induce fatigue in both steel and CFRP, with the bond between materials degrading under cyclic shear and normal stresses, reducing the overall load-bearing capacity and ductility of the column.
- Combined Loading**: Multi-axial loading can induce a complex stress state within the column, leading to more pronounced local effects and an increased risk of CFRP delamination or rupture. These loading conditions can alter the critical load for local and overall buckling, affecting stability and load-bearing capacity. Cyclic loading can also contribute to material fatigue, further impacting long-term performance.

By acknowledging these limitations and suggesting future research avenues, we aim to provide readers with a more comprehensive understanding and offer guidance for subsequent studies. The specific revisions in the manuscript are as follows:

[Section 7, page 32, lines 550 ~ 554] A new section has been added to the "Conclusion" to explicitly address these limitations and suggest future research directions.

The following text was included:

" Further research is needed to investigate the mechanical behavior of CFRP-CHST short columns under bending and cyclic loading. This includes analyzing the impact of CFRP reinforcement on bending stiffness and flexural strength, as well as studying the hysteretic behavior and fatigue life under dynamic loading, such as during earthquakes. These studies would provide a comprehensive basis for seismic and wind resistance design."

Comment 8:

The study is focused on circular CFRP-CHST columns, limiting the generalizability of the conclusions to non-circular cross-sections. Acknowledge this limitation and suggest extending the study to non-circular cross-sections. Discuss how the two formulas might differ for other geometries and propose a methodology for investigating these cases.

Response 8:

Thank you for raising the important issue regarding the limitations and potential extensions of the research. We fully acknowledge that our study primarily focuses on circular short columns, and we recognize that this limits the generalizability of our findings to non-circular cross-sections. We will explicitly address this limitation in the revised manuscript and indicate that future research should extend to non-circular cross-sections. Below is our detailed response to this issue:

**1. Research Limitations**

In this study, we concentrated on the axial compression behavior and failure mechanisms of circular CFRP-confined high-strength steel tube (CFRP-CHST) short columns. This focus was chosen due to the widespread use of circular cross-sections in structural engineering. However, we acknowledge that this choice limits the applicability of our conclusions to non-circular cross-sections, such as square or rectangular hollow steel tubes.

**2. Future Research Directions**

Circular cross-sections are common in civil engineering due to their uniform stiffness in all directions. However, in practical applications, structural components may feature rectangular, square, or other shapes. To extend the applicability of our research, we suggest that future studies explore non-circular cross-sections, such as rectangular hollow steel tubes (RHST) and square hollow steel tubes (SHST). These shapes are commonly used in structural engineering and may display different mechanical behaviors compared to circular cross-sections.

**3. Discussion on Formula Differences for Different Geometries**

For non-circular cross-sections, the effect of CFRP confinement may differ from that observed in circular cross-sections, leading to variations in the formulas for yield and ultimate load-bearing capacities. These differences can be attributed to several factors:

- Stress Distribution: Non-circular cross-sections exhibit different stress distributions under axial compression, which can affect the effectiveness of CFRP confinement. Stress may be uneven, especially at corners or edges, influencing the material's yield and ultimate strength.
- Local and Overall Buckling: Non-circular cross-sections may experience more complex buckling behaviors, potentially altering the impact of CFRP confinement on the ultimate load-bearing capacity. For instance, rectangular cross-sections may be more susceptible to local buckling at the corners, reducing their ultimate capacity.
- CFRP Wrapping Efficiency: The CFRP wrapping process on non-circular cross-sections may be uneven, affecting the confinement effectiveness. Non-circular cross-sections may require different wrapping techniques to ensure optimal confinement performance.

**4. Research Methodology**

To explore these scenarios, we propose the following methodology for future investigations:

- Theoretical Analysis: Develop theoretical models that account for the influence of non-circular cross-sections on stress distribution and buckling behavior. These models should address the effects of shape on local buckling and stress distribution.
- Finite Element Simulation: Use finite element analysis to simulate the behavior of CFRP-confined short columns with different cross-sectional shapes under axial compression. This will include material and geometric nonlinearity to assess variations in CFRP confinement effectiveness.
- Experimental Validation**: Conduct experimental studies to validate the theoretical models and numerical simulations, gathering performance data for CFRP-confined high-strength steel tube short columns with different cross-sectional shapes.
- Parameter Sensitivity Analysis: Perform a parameter sensitivity analysis to identify key factors influencing the performance of CFRP-confined short columns with non-circular cross-sections. This analysis would help evaluate the impact of parameter variations on structural performance.

We greatly appreciate your valuable feedback and will include a discussion of this limitation and the proposed future research directions in the revised manuscript. The specific revisions in the manuscript are as follows:

[Section 7, page 32, lines 555 ~ 560] A new section has been added to the "Conclusion" to explicitly address these limitations and suggest future research directions.

The following text has been included:

" Future studies should expand to include CFRP-confined steel tube short columns with non-circular cross-sections (e.g., square, rectangular, and elliptical). Research could focus on developing theoretical models for these shapes, conducting experimental tests on their performance under various loading conditions, and validating the models through numerical simulations to explore stress-strain distributions and failure modes. This research would support the optimized design of such components in practical engineering, improving safety and cost-effectiveness."

Comment 9:

More discussion about the effect of CFRP effect with higher slenderness ratios is required.

Response 9:

Thank you for your valuable suggestion. We acknowledge that analyzing the impact of CFRP confinement on short circular CFRP-CHST columns with high slenderness ratios is crucial. High slenderness ratio structures are common in practical engineering, and understanding the role of CFRP in such cases is vital for structural design and reinforcement. We agree that a detailed discussion of CFRP confinement effects under high slenderness ratios would significantly enhance the depth of our study and provide more comprehensive guidance for engineering applications. This aspect will be addressed in our future research. Below is our response:

**1. The Potential Impact of High Slenderness Ratios on CFRP Confinement Effects**

- Changes in Buckling Behavior For columns with high slenderness ratios, the likelihood of buckling increases. CFRP confinement can improve the local buckling strength of short columns but has a lesser impact on overall buckling resistance. Therefore, for columns with high slenderness ratios, the CFRP reinforcement effect may not be as pronounced as in columns with lower slenderness ratios. CFRP confinement significantly enhances buckling resistance by providing lateral support, helping delay the onset of both local and overall buckling.
- CFRP Stress Distribution: In short columns with high slenderness ratios, the stress distribution of CFRP along the column length may be uneven. In particular, the central region may experience lower stress in the CFRP, which could affect its reinforcement efficiency. The application of CFRP results in stress redistribution within the steel tube. As the slenderness ratio increases, the stress concentration in the critical buckling region can be alleviated by the CFRP fabric, which has high tensile strength and can carry part of the load.
- CFRP Layers and Layout: For high slenderness ratio columns, additional CFRP layers or a specific layout may be needed to enhance the load-bearing capacity and ductility. For example, applying more CFRP layers to the central region of the column may strengthen the local buckling resistance in that area.
- Applicability of the CFRP Confinement Factor: For high slenderness ratios, the CFRP confinement factor may require adjustment to more accurately reflect the confinement effect. This could be determined through experimental testing and numerical simulations. While CFRP confinement enhances the performance of high slenderness ratio columns, there are inherent limitations.

**2. Future Research Directions**

- Experimental Research: Conduct experimental studies on high slenderness ratio CFRP-CHST short columns to assess the actual effect of CFRP and evaluate the applicability of the CFRP confinement factor.
- Numerical Simulation: Use finite element analysis to model CFRP-CHST short columns under varying slenderness ratios, focusing on CFRP stress distribution and changes in the buckling modes of the columns.
- Improvement of Theoretical Models: Based on experimental and numerical results, refine theoretical models to predict the yield and ultimate load-bearing capacity of CFRP-CHST short columns with high slenderness ratios more accurately.

To address your suggestions, we will clarify the limitations of the study, especially concerning slenderness ratios, in the revised manuscript and propose future research directions. The specific revisions are as follows:

[Section 7, page 32, lines 561 ~ 565] In the revised manuscript, we have added a section in the "Conclusion" to explicitly address these limitations and propose future research. The following text has been added:

“Conducting an in-depth study of the mechanical behavior of CFRP-confined steel tube components with high slenderness ratios. This includes developing theoretical models for short columns with high slenderness ratios, conducting experimental studies to assess the performance of such columns under various loading conditions, and validating theoretical models through numerical simulations to investigate stress-strain distributions and failure paths. This research will improve the safety and cost-effectiveness of slender and long-span structures.”

Comment 10:

The references cited are largely irrelevant to the research topic, especially references from [1] to [10]. It is crucial that these references be updated to include more recent and pertinent sources that directly relate to the subject matter.

Response 10:

Thank you for your valuable comments. In response to the issues you raised, we have reviewed the referenced literature, particularly references [1] to [10], and replaced those that are not directly relevant to the research topic. In the revised manuscript, we have cited more recent studies that focus on the axial compression behavior and failure mechanisms of CFRP-CHST short columns. The specific revisions in the manuscript are as follows:

[Section 1, pages 3~ 5, lines 27~91; References, pages 35~ 37] The introduction section has been rewritten, irrelevant references have been replaced, and literature from the past three years has been incorporated.

We kindly request your esteemed review of the revised manuscript at your earliest convenience. We sincerely hope that these changes meet your expectations for publication, and we await your final evaluation of our revised manuscript. Should you have any additional comments or suggestions, we stand ready to address them promptly.

Thank you for your time and consideration.

Best regards,

Jian Chen, Hairong Huang, Yun Zhou, Kan Liu

2. Response to Reviewer #2

General comments:

From my perspective, this paper is highly repetitive and lacks novelty. The results presented are merely reiterations of existing findings that have been previously collected, analyzed, and modeled by our team through both numerical and machine-learning approaches. Unfortunately, there is no significant new contribution in this study. Extensive literature already covers similar numerical and theoretical work on these types of specimens, and existing models adequately address stress-strain relationships for these materials. Given this context, I do not believe this paper meets the standards for publication in any scholarly journal.

Response:

Dear Reviewer,

We deeply appreciate your time and effort in reviewing our manuscript and providing insightful feedback. We also commend your significant contributions to advancing research in this field. Your critical evaluation is invaluable, and we have carefully addressed your comments to enhance the quality and impact of our study, ensuring it meets the rigorous standards for publication. While we acknowledge the issues raised, we believe there may have been some misunderstandings regarding the scope and contributions of our research. Our study presents innovative findings in theoretical formula derivation and failure mechanism analysis, which were not sufficiently highlighted in the original manuscript. Below, we provide a detailed response to your comments to clarify how our work builds upon existing studies and contributes to advancing the field:

**1. Regarding the Issue of Reproducibility**

You noted that the manuscript exhibits high repetition, suggesting the results reiterate findings already collected, analyzed, and modeled through numerical and machine-learning methods. However, our study represents a significant advancement based on prior research, as outlined below:

- Distinct Research Focus. Existing studies have primarily concentrated on numerical and theoretical analysis of similar specimens. In contrast, our study investigates the behavior and failure mechanisms of CFRP-CHST short columns under axial compression. Our research emphasizes the effect of CFRP confinement on circular hollow steel tube (CHST) short columns, addressing gaps in the literature by exploring mechanical behavior at both the yield and ultimate states.
- Innovative Theoretical Formulas. We developed new theoretical formulas to calculate the yield and ultimate load-bearing capacities of CFRP-CHST short columns under axial loading. These formulas, derived using continuum mechanics and the limit equilibrium method, account for material properties, geometric parameters, and loading conditions. Notably, we introduced formulas for yield and ultimate confinement stress, providing results that align closely with real-world engineering conditions.
- Introduction of the CFRP Confinement Factor. Our study introduces the CFRP confinement factor, which quantifies the enhancement in load-bearing capacity within the effective range of CFRP confinement. This metric offers guidance for optimizing the number of CFRP layers in engineering design, improving reinforcement efficiency, and reducing material waste. Previous studies have not proposed similar quantitative metrics.

**2. Regarding the Issue of Innovation.**

While acknowledging existing literature on numerical and theoretical analysis of CFRP-confined steel columns, our study offers the following novel contributions:

- Development of New Theoretical Formulas. We derived comprehensive formulas for yield and ultimate strength, confinement stress, and axial displacement under different states. These formulas provide practical methods for designing and verifying CFRP-CHST short columns, addressing limitations in existing models and enhancing engineering applications.
- Detailed Analysis of Failure Mechanisms. Through theoretical analysis, finite element simulation, and experimental validation, we examined the effects of CFRP confinement on failure modes. Our findings reveal that the ultimate load-bearing capacity increases with additional CFRP layers, but excessive confinement alters failure modes from local bulging to inward buckling. These insights provide critical guidance for designing effective reinforcement schemes.
- Integration of Numerical and Experimental Validation. The study validates theoretical formulas through finite element analysis and comparison with experimental data, ensuring reliability and applicability. This integration strengthens the study’s contribution to both theoretical and practical advancements.
- Practical Guidance for Engineering Applications. The research offers valuable design methods for CFRP reinforcement, including the introduction of the CFRP confinement factor. This metric optimizes reinforcement design reduces material waste, and enhances structural performance and safety.

**3. Regarding the Issue of Literature Coverage**

You expressed concerns about the existing body of literature addressing similar topics. While acknowledging previous achievements, our study addresses critical gaps:

- Limited Research on Metal Structures. While CFRP reinforcement has been extensively studied in concrete structures, research on its application to CHST short columns remains sparse. Our study provides a theoretical foundation and methods specifically for metal structures.
- Inadequate Exploration of Failure Mechanisms. Previous research has not systematically investigated failure mechanisms in CFRP-reinforced CHST short columns. Our study identifies the limitations of CFRP reinforcement and enhances understanding of buckling deformation under varying conditions.
- Lack of Design Methods. Current design approaches for CFRP-reinforced CHST short columns are insufficiently accurate and practical. Our study introduces novel design methods, including theoretical formulas and the CFRP confinement factor, improving design efficiency and precision.

This study offers significant contributions through the development of new theoretical formulas, detailed failure mechanism analysis, integration of numerical and experimental validation, and practical guidance for engineering applications. We have made substantial revisions to the manuscript to emphasize these contributions and address your concerns.

Once again, we sincerely thank you for your thorough review and valuable comments. We hope the revisions and clarifications provided demonstrate the manuscript’s suitability for publication.

We kindly request your esteemed review of the revised manuscript at your earliest convenience. We sincerely hope that these changes meet your expectations for publication, and we await your final evaluation of our revised manuscript. Should you have any additional comments or suggestions, we stand ready to address them promptly.

Thank you for your time and consideration.

Best regards,

Jian Chen, Hairong Huang, Yun Zhou, Kan Liu

3. Response to Reviewer #3

General comments:

This manuscript has taken CFRP-CHST short columns into consideration and studied the structural behaviour of such columns under axial compression. The study is well organized and the authors tried to investigate the concept of their study through different methods. While the novelty of the study is a bit poor, the package of the whole study is considerable from my point of view. However, it needs some amendments to fulfill the requirements of publishing in the journal.

Response:

Dear Reviewer,

We sincerely appreciate your positive feedback on our manuscript, particularly your recognition of the organization of our research and the application of methods. Your encouraging comments have been highly motivating for us. In response to your suggestions for revisions to meet the journal's publication standards, we have carefully reviewed and implemented the proposed changes. All of your suggestions have been fully accepted, and the corresponding revisions have been incorporated into the updated manuscript.

Comment 1:

*Lines 106-109: This part needs to be polished in terms of language.*

Response 1:

Thank you for identifying areas for improvement in the manuscript. In response to your comments on lines 106 to 109, we have carefully revised and refined this section to enhance clarity and professionalism. The updated expression in the revised manuscript is as follows:

[Section 2, Page 7, lines 116 ~ 119] The corresponding expression in the original manuscript has been revised to: “The circumferential confinement force provided by CFRP is denoted as $q_{f}$, with the thickness of the steel tube represented by $t_{s}$, the inner diameter by $d$, and the outer diameter by $D=d+2t_{s}$. The yield strength is denoted as $f_{y}$. The force schematic for the steel tube and CFRP is illustrated in Fig. 2.”

Comment 2:

*Fig. 2: The stress distribution is shown separately for the steel tube and the CFRP. Since I have seen this type of illustration a lot, I suggest showing the stress distribution on a section of CFRP-CHST. Can you do it?*

Response 2:

Thank you for the valuable suggestion. A more intuitive stress distribution diagram for the CFRP-CHST cross-section has been incorporated to better illustrate the interaction and stress transfer between the steel tube and CFRP. In response, Figure 2 in the original manuscript has been revised to present a comprehensive depiction of stress distribution across the CFRP-CHST cross-section. The specific revisions in the revised manuscript are summarized as follows:

[Figure 2, page 7] The updated Figure 2 now provides a clear visualization of the stress distribution within the entire cross-section, including the steel tube and the CFRP layer.

For ease of review, the revised Figure 2 from the original manuscript is presented in Fig. R2- 1.

Fig. R2- 1 Stress analysis of CFRP-CHST short columns: (a) stress distribution on a section of CFRP-CHST, (b) stress state of the reinforced steel tube, and (c) stress state of CFRP.

[Title, Page 1] XXX

Comment 3:

*For the equations you need to clarify each parameter beneath the formula.*

Response 3:

Thank you for your suggestion. We fully agree that providing a clear explanation of each parameter below the formula is crucial for enhancing the readability and understanding of the paper. In the revised manuscript, we will provide detailed explanations for all the parameters in the formulas. By explaining each parameter in this manner, we aim to help readers better understand the physical meaning and application context of the formulas. We have provided detailed explanations for each parameter involved in the formulas. The specific revisions in the revised manuscript are as follows:

[Nomenclature, pages 2~ 3; Section 2, pages 5 ~ 12] In the revised manuscript, all abbreviations and symbols are defined upon their first appearance. Additionally, a list summarizing the symbols used has been included. Specifically, we have made detailed additions. First, we have included a parameter list that explains the meaning and units of each variable and parameter. A section titled "Nomenclature" has been added before the introduction, providing clear definitions for all abbreviations and acronyms used throughout the manuscript. Additionally, below each formula, we have provided a brief description of each parameter, including its physical significance and its role in the formula.

Comment 4:

*Equations 5 and 18: It seems the authors just considered the area of steel. What about the impact of carbon fiber reinforced polymer? Has been ignored?*

Response 4:

Thank you for the insightful observation regarding the consideration of CFRP in Eq. (5) and Eq. (18). The original manuscript lacked clarity, and revisions have been made to address this issue. Both equations account for the contributions of the steel tube and CFRP, incorporating the stress and strain of the CFRP layers and their interaction with the steel. As illustrated in Fig. R2-1(b), CFRP, due to its high tensile strength and low compressive strength, does not directly resist axial compression but instead provides lateral confinement to the steel tube. This confinement enhances the yield and ultimate bearing capacity of the composite structure. The specific contributions of CFRP to the bearing capacity in these equations are explained as follows:

**1. Contribution of CFRP in Eq. (5):**

Eq. (5) from the manuscript is as follows:

$N=\sigma_{\text{zs}}A_{\text{s}}$ (5)

In this equation, CFRP contributes to the bearing capacity by providing lateral confinement to the steel tube. The stress and strain in the CFRP layers are explicitly considered, and their confinement effect prevents premature local buckling, improving the composite behavior of the steel and CFRP system. The axial stress of the steel tube, denoted as $\sigma_{\text{zs}}$, includes the influence of the CFRP's lateral confinement stress $q_{\text{f}}$. The confinement stress $q_{\text{f}}$ is determined by the material properties of the CFRP, such as tensile strength and elastic modulus, as well as its geometric properties, including thickness and the number of layers. These factors collectively determine the degree of lateral confinement, enhancing the overall axial stress and bearing capacity of the CFRP-CHST system.

$\left\{\begin{matrix} \sigma_{\text{zs}}=\sqrt{f_{\text{y}}^{2}-\frac{3}{4}\left(\sigma_{\text{θs}}-\sigma_{\text{rs}}\right)^{2}}+\frac{\sigma_{\text{θs}}+\sigma_{\text{rs}}}{2} \\ \int_{d/2}^{d/2+t_{\text{s}}} \sigma_{\text{θs}}d\rho=\frac{q_{\text{f}}d}{2} \\ \int_{d/2+t_{\text{s}}}^{d/2+t_{\text{s}}+t_{\text{eq,f}}} \sigma_{\text{θf}}d\rho=\frac{-q_{\text{f}}\left(d+2t_{\text{s}}\right)}{2} \\ \sigma_{\text{rs}}=q_{\text{f}} \end{matrix}\right.$ (R3-1)

**2. Contribution of CFRP in Eq. (18)**

Eq. (18) from the manuscript is as follows:

$N_{\text{sf,y}}=\left(\sqrt{1-\frac{3}{4}\left[\left(\beta-1\right)\psi\right]^{2}}+\frac{\left(\beta+1\right)\psi}{2}\right)A_{\text{s}}f_{\text{y}}$ (18)

CFRP's contribution to this equation is also reflected through its lateral confinement effect, which is expressed by confinement stress dependent on both material properties (tensile strength and modulus of elasticity) and geometric properties (thickness and number of CFRP layers). This confinement enhances the steel tube's resistance to axial buckling, increasing the overall load-carrying capacity. Parameters $\psi$, $\beta$, and $\gamma$ reflect the combined contributions of the CFRP and steel tube. Specifically:

- $\psi$: Represents the combined effect of material and geometric properties of the steel and CFRP on the confinement.
- $\gamma$: Depends on the outer diameter of the steel tube and the equivalent thickness of CFRP, quantifying the lateral confinement provided by the CFRP.

These parameters describe the role of CFRP in enhancing the stability and axial load capacity of the CFRP-CHST system.

$\left\{\begin{matrix} \psi=\frac{\mu_{\text{s}}}{\sqrt{\left(\frac{E_{\text{s}}\gamma}{E_{\text{f}}}-\beta+\frac{\mu_{\text{s}}\left(\beta+3\right)}{2}\right)^{2}+\frac{3}{4}\mu_{\text{s}}^{2}\left(\beta-1\right)^{2}}} \\ \beta=\frac{d}{2t_{\text{s}}} \\ \gamma=\frac{D}{2t_{\text{eq,f}}} \end{matrix}\right.$ (R3-1)

Although the direct contribution of CFRP (Expressed in terms of the area of CFRP) to the yield-bearing capacity is not explicitly detailed in these equations, both equations consider the combined effects of the steel tube and CFRP. The confinement effect of CFRP is a key factor influencing the load-carrying capacity of the system.

To address your concerns, we have made the following revisions to the revised manuscript:

[Section 2, Page 8, lines 143 ~ 145; Section 2, Page 10, lines 185 ~ 187] In the revised manuscript, we have indicated the contribution of CFRP below the corresponding equations.

The text below Eq. (5) has been updated to state: "In Eq.(5), the axial stress of the steel tube is influenced by the stress-strain behavior of CFRP. The confining effect of CFRP enhances the buckling resistance and stability of the steel tube, thereby improving the bearing capacity of the component."

The text below Eq. (18) has been updated to state: "In Eq. (18), the parameter ψ is determined by the geometric and material properties of both the steel tube and CFRP. The confinement provided by CFRP enhances the yield-bearing capacity of the CFRP-CHST short column."

Comment 5:

*Line 136: What is assumption 2? Please explain.*

Response 5:

Thank you for your inquiry. The original manuscript, on Page 5, provides a theoretical analysis containing three assumptions, which are located in lines 96–97. Assumption 2 posits that " *The bond between the steel tube and CFRP is assumed to be fully intact, with no separation occurring during loading, ensuring collaborative deformation of both materials* [1,2]." In our study, this assumption implies that there is no relative slippage between the steel tube and the CFRP during loading, and the bond between the two remains entirely intact. This assumption is grounded in the following considerations:

**1. Synergistic deformation between CFRP and the steel tube**

We assume that the bond strength between the CFRP and steel tube is sufficiently high to ensure that both materials deform together during axial compression, without relative movement between them. This guarantees that the circumferential restraint provided by the CFRP is effectively transmitted to the steel tube. Under this assumption, the steel tube and CFRP function as a unified system, jointly bearing the external load. This synergistic behavior is essential for evaluating the reinforcing effect of CFRP on the steel tube.

**2. Experimental data support**

Numerous experiments and field applications typically use specific adhesives to ensure a strong bond between CFRP and steel cylinders [1,2]. Thus, this assumption is generally valid in practical scenarios.

To address your concerns, we will provide a more detailed clarification of this assumption in the relevant section of the manuscript, ensuring that readers fully understand its basis and implications for the analysis. The revisions in the updated manuscript will be as follows:

[Section 2, Pages 6~7, lines 105–111; Section 3, Pages 16~17, lines 307–312] The three assumptions presented in the original manuscript are as follows: “Based on experimental results [1,2], several reasonable computational assumptions are proposed as follows: (1) CFRP fabric is treated as a linearly elastic material, with tensile strength only in the fiber direction, while other mechanical properties are disregarded. (2) The bond between the steel tube and CFRP is assumed to be fully intact, with no separation occurring during loading, ensuring collaborative deformation of both materials [1,2]. (3) Steel is considered an isotropic material. For simplification and clarity in the bearing capacity calculation, the steel tube is assumed to behave as an ideal elastic-plastic material, following the Von Mises yield criterion.”. In addition, Section 3 provides a detailed discussion of the rationale behind this assumption and the approach used in the numerical model.

[1]	J. Haedir, X.-L. Zhao, Design of short CFRP-reinforced steel tubular columns, Journal of Constructional Steel Research 67 (2011) 497–509. https://doi.org/10.1016/j.jcsr.2010.09.005.

[2]	J.G. Teng, Y.M. Hu, Behaviour of FRP-jacketed circular steel tubes and cylindrical shells under axial compression, Construction and Building Materials 21 (2007) 827–838. https://doi.org/10.1016/j.conbuildmat.2006.06.016.

Comment 6:

*Lines 124, 126, 168: The terms of bearing capacity, yield bearing capacity, ultimate bearing capacity were used and the final equations 5, 18, 22 are a bit confusing! You can provide and highlight a final formula as the outcome of this study. I think would be user-friendly.*

Response 6:

Thank you for your feedback. In response to the issue raised, the authors provide the following explanation:

The theoretical formulas for the bearing capacity of CFRP-CHST short columns are categorized as yield-bearing capacity and ultimate bearing capacity, which correspond to the yield and ultimate states of the specimens, respectively. Equation (5) represents an intermediate step in the theoretical derivation, while Equation (18) calculates the yield-bearing capacity, and Equation (22) calculates the ultimate bearing capacity. To enhance the clarity and usability of the research findings, we acknowledge the need to provide a clear final formula and ensure that these formulas are prominently presented and easy to understand. Therefore, in response to your concerns, we have emphasized the final formula after the theoretical derivation.

The specific revisions in the revised manuscript are as follows:

[Section 2, Page 12, lines 218 ~ 225] The revised manuscript now includes the following additions:

"In summary, the bearing capacity of CFRP-CHST short columns is categorized into yield-bearing capacity and ultimate bearing capacity, corresponding to the yield and ultimate states of the component, respectively. Eq. (18) can be used to calculate the yield-bearing capacity of the CFRP-CHST short column, while Eq. (22) can be used to calculate the ultimate bearing capacity of the CFRP-CHST short column. These two equations provide reference methods and practical tools for the bearing capacity design of CFRP-CHST short columns in the yield and ultimate states."

Comment 7:

Equations 5, 18, and 22: Still, I could not recognize the area of CFRP in these equations. Please clarify.

Response 7:

Thank you for your continued attention to the details of our equations, particularly regarding the representation of the carbon fiber reinforced polymer (CFRP) area. As addressed in our response to "Comment 4," the area of CFRP is not directly included in the formula because CFRP does not bear axial load. Instead, the contribution of CFRP to the bearing capacity is determined by the hoop confinement stress. In other words, the three equations simultaneously account for the contributions of both the steel tube and the carbon fiber fabric. As shown in Figure R2-1(b), while CFRP resists tensile stress, it does not resist compressive stress and thus does not directly bear the axial load. Instead, CFRP provides hoop confinement to the steel tube, enhancing both the yield and ultimate bearing capacities through this confinement effect.

In Equation (5), the contribution of CFRP is reflected in its lateral confinement of the steel tube. The stress and strain in the CFRP layers are considered, and the confinement effect increases the steel tube's resistance to buckling. Since CFRP does not directly carry axial loads, its primary role is to enhance the overall structural performance by preventing premature local buckling and improving the composite behavior of the steel and CFRP system. This confinement effect is modeled in the formula by a term that accounts for the stress in the CFRP and its interaction with the steel tube.

In Equation (18), CFRP contributes to the yield-bearing capacity through its lateral confinement effect on the steel tube, represented by the confinement stress. This stress depends on both the material properties of the CFRP (such as tensile strength and modulus of elasticity) and its geometric properties (such as thickness and number of layers). By providing lateral support, CFRP enhances the steel tube's resistance to axial buckling, increasing the composite structure's yield-bearing capacity. The interaction between the CFRP and steel tube is captured by including the confinement stress, which directly influences axial deformation and stability.

Equation (22) calculates the ultimate bearing capacity of CFRP-CHST short columns, considering the contributions of both the steel tube and CFRP. This equation explicitly incorporates the tensile strength and reinforcement thickness of the CFRP, with the area of CFRP calculable based on its reinforcement thickness. The final formulas provided are simplified to facilitate practical application in engineering design.

To address your concerns, the revised manuscript will include a clear explanation of how CFRP affects the yield and ultimate bearing capacity of CFRP-CHST short columns. The specific revisions are as follows:

[Section 2, Page 8, lines 143–145; Page 10, lines 185–187; Page 12, lines 218–219]

- The text " In Eq.(5), the axial stress of the steel tube is influenced by the stress-strain behavior of CFRP. The confining effect of CFRP enhances the buckling resistance and stability of the steel tube, thereby improving the bearing capacity of the component." has been added in lines 143–145.
- The text "In Eq. (18), the parameter ψ is determined by the geometric and material properties of both the steel tube and CFRP. The confinement provided by CFRP enhances the yield-bearing capacity of the CFRP-CHST short column." has been added in lines 185–187.
- The text " As shown in Eq. (22), the tensile strength of CFRP ($f_{f}$) and the reinforcement thickness ($t_{\text{eq,f}}$ ) have a significant impact on the ultimate bearing capacity of the CFRP-CHST short column." has been added in lines 218–219.

Comment 8:

Table 1: What are nl , nh ?

Response 8:

Thank you for highlighting the need to clarify the symbols $n_{L}$ and $n_{H}$ in Table 1, as this is essential for improving both understanding and readability. These two symbols represent different CFRP layer configurations. Specifically, $n_{H}$ denotes the number of CFRP layers in the hoop direction, while $n_{L}$ represents the number of CFRP layers in the longitudinal direction. In the experimental study by Haedir and Zhao [1,2], CFRP was applied to CHST short columns in alternating hoop and longitudinal layers to achieve optimal confinement effects. To enhance clarity, the following revisions have been made to the revised manuscript:

[Nomenclature, Pages 2~ 3; Section 2, page 7, lines 125 ~ 128; Table 1]

- Nomenclature (Pages 2–3): A list titled "Nomenclature" has been added before the introduction, providing precise definitions for all abbreviations and acronyms used throughout the text.
- Section 2, Page 7, Lines 125–128: An explanatory note has been added at the first instance where these parameters appear in the manuscript.
- Table 1: A footnote has been added below Table 1 to explicitly clarify the meanings of $n_{L}$ and $n_{H}$.

These modifications aim to ensure that all parameters in the manuscript are clearly defined and easily understood by readers.

[1]	J. Haedir, X.-L. Zhao, Design of short CFRP-reinforced steel tubular columns, Journal of Constructional Steel Research 67 (2011) 497–509. https://doi.org/10.1016/j.jcsr.2010.09.005.

[2]	J.G. Teng, Y.M. Hu, Behaviour of FRP-jacketed circular steel tubes and cylindrical shells under axial compression, Construction and Building Materials 21 (2007) 827–838. https://doi.org/10.1016/j.conbuildmat.2006.06.016.

Comment 9:

*Table 2: I think it is unnecessary as the necessary details of CFRP are already mentioned in Section 3.1.*

Response 9:

Thank you for your valuable feedback. To enhance the conciseness of our paper and eliminate redundant information, we have removed Table 2 and revised Section 3.1 to more effectively present the material parameters. These changes ensure that all content is necessary, relevant, and presented most efficiently. The specific revisions in the revised manuscript are as follows:

[Section 3, Page 14, lines 258 ~ 270] The content of Table 2 has been fully integrated into Section 3 in text form, ensuring that the material parameters are presented clearly and without redundancy.

Comment 10:

Line 253 needs a space.

Response 10:

Thank you for bringing the formatting issues to our attention. We will conduct a thorough review of the entire manuscript to ensure that no similar formatting problems remain and that the document meets the required standards.

Comment 11:

Fig 4: needs some adjustments. One arrow is missing and b) overlaps with the column.

Response 11:

Thank you for your detailed feedback on Figure 4. We have made the necessary revisions to enhance the clarity and accuracy of the figure. The changes in the revised manuscript are as follows:

[Figure 4, page 16] The figure has been updated to improve clarity, ensuring that all labels, axes, and data points are visible and correctly represented.

To facilitate the review process, the revised version of Figure 4 is provided in Fig. R3- 1.

Fig. R3- 1 CFRP material orientation definitions: (a) circumferential, and (b) longitudinal.

Comment 12:

Table 3: The information here can be written within the manuscript and there is no need for a single-row Table here.

Response 12:

Thank you for your suggestion. We agree that incorporating the information from Table 3 directly into the manuscript body eliminates the need for a separate single-line table. This approach enhances conciseness and better integrates the data into the article’s narrative. The information from Table 3 has been incorporated into the relevant sections of the manuscript, ensuring smooth integration with the surrounding text. The specific revisions in the revised manuscript are as follows:

[Section 3, Page 14, lines 258 ~ 270; Page 16, lines 298 ~ 306] The material property test data for the fibers are now presented in textual form in lines 258 ~ 270. The data previously in Table 3 are now provided in textual form in lines 298 ~ 306.

Comment 13:

I think there is no need for 3 sub-sections for Section. 3 as all of the subsections are very short.

Response 13:

Thank you for your suggestion regarding the structure of Section 3. We acknowledge your observation that the three subsections were brief and agree that merging them can enhance the coherence and readability of the manuscript. Accordingly, we have made the necessary adjustments to Section 3 to ensure clarity, conciseness, and efficient communication of our approach. The specific revisions in the revised manuscript are as follows:

[Section 3, Pages 14~17] The three brief subsections have been merged into a more cohesive section, eliminating redundancy and improving the flow of information. The methodology is now presented in a more streamlined and organized manner, ensuring that key points are effectively conveyed. The revised section has been restructured to enhance readability and accessibility for the reader.

Comment 14:

Line 292: Do you mean Yield?

Response 14:

Thank you for your thorough review of the manuscript. You correctly identified that the line likely refers to the concept of "yield." Accordingly, we have made the necessary revisions in the updated manuscript. The specific changes are as follows:

[Section 4.2.1, Page 19, line 341] The term has been updated to "yield" to more accurately convey the intended concept. Additionally, we have reviewed the manuscript to ensure consistent use of terminology related to yield and other related concepts.

Comment 15:

Line 301: “Comparison of longitudinal displacement at yield for CHST short columns”, at yield level/point.

Response 15:

Thank you for highlighting the need to clarify the expression "Comparison of longitudinal displacement at yield for CHST short columns" in line 301, which should refer to the yield point or yield level. To enhance accuracy and clarity, we will revise this line to explicitly indicate that the comparison is made at the yield point. The revised content is as follows:

[Section 4.2.1, Page 19, line 350] The original phrase "Comparison of longitudinal displacement at yield for CHST short columns" has been updated to "Comparison of longitudinal displacement at the yield point for CHST short columns." Furthermore, we have thoroughly reviewed the manuscript to ensure the accuracy and consistency of all similar expressions.

Comment 16:

Table 5: It needs a footnote explaining the parameters.

Response 16:

Thank you for your suggestion. We agree that adding footnotes to Table 5 to clarify the parameters is essential for helping readers better understand the data. Consequently, we have included detailed footnotes below Table 5 (which corresponds to Table 3 in the revised manuscript) to explain each parameter. The specific revisions in the revised manuscript are as follows:

[Table 3, Page 24, Lines 418–423] Footnotes have been added below Table 3 to provide definitions and descriptions for each parameter used in the table, ensuring clarity and enhancing the reader’s understanding.

To facilitate the review process, the footnotes added after Table 5 are provided below:

“Note: $t_{s}$ is the thickness of the steel tube, $N_{\text{s,y}}$ is the yield-bearing capacity for CHST short columns, $q_{\text{f}\text{,y}}$ is radial stress of the steel tube at the yield state, $N_{\text{sf,y}}$ is the theoretical value of the yield-bearing capacity of CFRP-CHST short columns, $W_{\text{sf,y}}$ is the improvement ratio of the yield load-bearing capacity, $N_{\text{FE,y}}$ is the finite element value of the yield load-bearing capacity of CFRP-CHST short columns, $q_{\text{f,l}}$ is radial stress of steel tube at the ultimate state, $N_{\text{sf,l}}$ is the theoretical value of the ultimate load-bearing capacity of CFRP-CHST short columns, $W_{\text{sf,l}}$ is the improvement ratio of the ultimate load-bearing capacity, $N_{\text{FE,l}}$ is the finite element value of the ultimate load-bearing capacity of CFRP-CHST short columns, $N_{\text{e,l}}$ is the experimental value of the ultimate load-bearing capacity of CFRP-CHST short columns.”

Comment 17:

Conclusion Line 6: “A theoretical formula “, please highlight which one.

Response 17:

Thank you for highlighting the need to specifically mention the "theoretical formula" and its relevant object in the first conclusion. In response to your suggestion, we have revised the conclusion to explicitly reference the key theoretical formulas developed in our study. As noted in our responses to Comments 4, 6, and 7, the theoretical formulas for the bearing capacity of CFRP-CHST short columns include those for yield-bearing capacity and ultimate bearing capacity, corresponding to the yield state and ultimate state, respectively. The specific revisions in the revised manuscript are as follows:

[Section 7, Page 31, Lines 523–528] The first conclusion has been revised to:

“(1) New theoretical formulas were developed to calculate the yield-bearing capacity (Eq. 18) and ultimate bearing capacity (Eq. 22) of CFRP-CHST short columns under axial compression, based on continuum mechanics and the limit equilibrium method. The validity of these formulas was confirmed through finite element simulations and experimental data. The average ratio of the calculated yield load to the simulation results was 1.01, with a standard deviation of 0.0227, and the ratio for the ultimate load was 1.03, with a standard deviation of 0.0210.”

Comment 18:

Conclusion No. 3: How this is concluded? From FEA?

Response 18:

Thank you for your inquiry regarding the third conclusion in our manuscript, particularly concerning the source of this conclusion. In response, we provide the following clarification:

The third conclusion addresses the limitations of the ultimate bearing capacity of CFRP-reinforced CHST short columns when the CFRP confinement factor exceeds a certain threshold. This conclusion is derived from theoretical analysis, numerical simulations (FEA), and a review of relevant experimental data.

First, our theoretical analysis, grounded in the principles of continuum mechanics and the limit equilibrium method, forms the basis for understanding the behavior of CFRP-CHST short columns under axial compression and the role of CFRP confinement in enhancing bearing capacity.

Second, finite element analysis (FEA) is crucial for validating our theoretical model. It allows us to simulate various scenarios, such as different numbers of CFRP layers and confinement factors. The FEA results provide insights into the column’s behavior under varying conditions and help identify the threshold beyond which additional CFRP layers no longer significantly enhance the ultimate bearing capacity.

Finally, we referenced experimental data from the literature to support our conclusions. These experiments offer empirical evidence of the performance of CFRP-CHST short columns, which is essential for validating our theoretical model and numerical simulations.

We appreciate your feedback and have revised the conclusion section to clearly specify the basis for this conclusion. The specific revisions in the revised manuscript are as follows:

[Section 7, Page 31, Lines 529–541] The second and third conclusions have been revised as follows:

“(2) Both theoretical and numerical analyses suggest that CFRP reinforcement has a limited effect on the yield-bearing capacity but significantly enhances the ultimate bearing capacity of CHST short columns, with some limitations. The yield load improvement due to CFRP reinforcement was minimal, with a maximum increase of only 3.86%. However, the enhancement in ultimate load was more pronounced, although the effect diminished as the number of CFRP layers increased. CFRP also improved the ductility of CHST short columns, which is advantageous for seismic strengthening. Thus, CFRP reinforcement shows substantial potential for seismic retrofitting applications.

(3) The failure modes of CFRP-CHST short columns were influenced by CFRP reinforcement, with the failure mode transitioning from localized outward bulging at the ends to inward buckling deformation as the number of CFRP layers increased. For specimens with identical CFRP configurations, thicker steel tubes exhibited localized end bulging, whereas thinner tubes showed inward buckling away from the ends. The failure mode shifted from outward bulging to inward buckling with an increase in CFRP layers.”

Comment 19:

I suggest to re-write the conclusions with specific details. The conclusion looks broad and not only for this study.

Response 19:

Thank you for your suggestions. We have revised the conclusions to ensure they are specific, precise, and directly reflect the primary findings and contributions of our study. Additionally, we have summarized the limitations of the study and proposed potential directions for future research. These revisions enhance the understanding of the behavior of CFRP-CHST short columns and broaden their applicability in engineering practices. The specific revisions in the revised manuscript are as follows:

[Section 7, Pages 30~32] The rewritten conclusions are as follows:

“A comprehensive study has been conducted on the mechanical performance and failure mechanisms of CFRP-reinforced CHST short columns under axial compression, utilizing theoretical research, numerical analysis, and experimental data from the literature. The key findings are as follows:

(1) New theoretical formulas were developed to calculate the yield-bearing capacity (Eq. 18) and ultimate bearing capacity (Eq. 22) of CFRP-CHST short columns under axial compression, based on continuum mechanics and the limit equilibrium method. The validity of these formulas was confirmed through finite element simulations and experimental data. The average ratio of the calculated yield load to the simulation results was 1.01, with a standard deviation of 0.0227, and the ratio for the ultimate load was 1.03, with a standard deviation of 0.0210.

(2) Both theoretical and numerical analyses suggest that CFRP reinforcement has a limited effect on the yield-bearing capacity but significantly enhances the ultimate bearing capacity of CHST short columns, with some limitations. The yield load improvement due to CFRP reinforcement was minimal, with a maximum increase of only 3.86%. However, the enhancement in ultimate load was more pronounced, although the effect diminished as the number of CFRP layers increased. CFRP also improved the ductility of CHST short columns, which is advantageous for seismic strengthening. Thus, CFRP reinforcement shows substantial potential for seismic retrofitting applications.

(3) The failure modes of CFRP-CHST short columns were influenced by CFRP reinforcement, with the failure mode transitioning from localized outward bulging at the ends to inward buckling deformation as the number of CFRP layers increased. For specimens with identical CFRP configurations, thicker steel tubes exhibited localized end bulging, whereas thinner tubes showed inward buckling away from the ends. The failure mode shifted from outward bulging to inward buckling with an increase in CFRP layers.

(4) The CFRP confinement coefficient was introduced to quantify the enhancement of bearing capacity in CFRP-reinforced CHST short columns. A reference value for the optimal number of CFRP layers was proposed. When the CFRP confinement coefficient exceeds 1.155, further increases in the number of CFRP layers do not significantly improve the ultimate bearing capacity. In practical applications, the number of CFRP layers should be optimized based on the CFRP confinement coefficient to improve efficiency and reduce material waste. It is recommended that the CFRP confinement coefficient not exceed 1.155 to balance strengthening effectiveness and material costs.”

Comment 20:

Line 25: In the submission process, it is mentioned that the data is available within the manuscript and supplementary information, and the statement “Data will be made available on request” contradicts this.

Response 20:

Thank you for highlighting the inconsistency regarding the data availability statement. All data in this study is available within the manuscript, including detailed results in tables and figures. No separate data request is necessary, as everything in the submitted document is accessible. We have revised this statement to ensure consistency with the information provided during the submission process. The specific revisions in the revised manuscript are as follows:

[Data availability, Page 32 The relevant statement has been revised to: "Data is available within the manuscript and supplementary information."

Comment 21:

The references are very limited and almost 10 of 23 are outdated. It is usually suggested to reference articles older than ten years for only 10 per cent of the total citations.

Response 21:

Thank you for your feedback on the references section. We understand the importance of citing the latest research to ensure the timeliness and relevance of the paper. The following measures have been taken: The reference list has been reviewed and updated to ensure that at least 90% of the citations are from studies published within the last ten years. This includes the latest experimental research, theoretical analyses, and numerical simulation results to reflect recent advancements in the field. A thorough literature search and review were conducted to incorporate the most recent publications directly related to our research topic. This includes newly published papers, review articles and the latest advancements in relevant fields. References older than ten years have been evaluated for their relevance. If they are essential for understanding the research background or theoretical foundation, they have been retained and their significance has been explicitly highlighted in the manuscript.

The specific revisions in the revised manuscript are as follows:

[Section 1, Pages 3–5; References, pages 35 ~ 37 ] The introduction has been extensively rewritten to ensure that all references are necessary, relevant, and presented effectively.

We kindly request your esteemed review of the revised manuscript at your earliest convenience. We sincerely hope that these changes meet your expectations for publication, and we await your final evaluation of our revised manuscript. Should you have any additional comments or suggestions, we stand ready to address them promptly.

Thank you for your time and consideration.

Best regards,

Jian Chen, Hairong Huang, Yun Zhou, Kan Liu
